# Necrosulfonamide Ameliorates Neurological Impairment in Spinal Cord Injury by Improving Antioxidative Capacity

**DOI:** 10.3389/fphar.2019.01538

**Published:** 2020-01-09

**Authors:** Jianhang Jiao, Yang Wang, Pengfei Ren, Shicai Sun, Minfei Wu

**Affiliations:** Department of Orthopedics, The Second Hospital of Jilin University, Changchun, China

**Keywords:** spinal cord injury, necrosulfonamide, mixed-lineage kinase domain-like protein activation, neurological impairment, antioxidative capacity

## Abstract

Currently, there is no efficient therapy for spinal cord injury (SCI). Anoxemia after SCI is a key problem, which leads to tissue destruction, while hypoxia after SCI induces cell injury along with inflammation. Mixed-lineage kinase domain-like protein (MLKL) is a critical signal molecule of necroptosis, and mitochondrial dysfunction is regarded as one of the most pivotal events after SCI. Based on the important role of MLKL in cell damage and potential role of mitochondrial dysfunction, our study focuses on the regulation of MLKL by Necrosulfonamide (NSA) in mitochondrial dysfunction of oxygen-glucose deprivation (OGD)-induced cell damage and SCI-mice, which specifically blocks the MLKL. Our results showed that NSA protected against a decrease in the mitochondrial membrane potential, adenosine triphosphate, glutathione, and superoxide dismutase levels and an increase in reactive oxygen species and malonyldialdehyde levels. NSA also improved the locomotor function in SCI-mice and OGD-induced spinal neuron injury through inhibition of MLKL activation independently of receptor-interacting protein kinase 3 (RIP3) phosphorylation. Besides the protective effects, NSA exhibited a therapeutic window. The optimal treatment time was within 12 h after the injury in the SCI-mice model. In conclusion, our data suggest a close association between the NSA level inhibiting p-MLKL independently of RIP3 phosphorylation and induction of neurological impairment by improving antioxidative capacity after SCI. NSA ameliorates neurological impairment in SCI through inhibiting MLKL-dependent necroptosis. It also provides a theoretical basis for further research and application of NSA in the treatment of SCI.

## Introduction

Spinal cord injury (SCI) represents a serious trauma to the central nervous system (CNS) and often causes a devastating impact on the motor function and many essential physiological functions (Courtine and Sofroniew, 2019). The main characteristics of SCI are its high incidence (22/100 million), high disability rate (about 37% of whole-body paralysis), high financial burden (from 50,000 to 70,000 dollars per patient every year), and that it primarily occurs in young people between 20 and 30 years old (Rossignol et al., 2007). Due to its complex and variable pathophysiology, there is still no effective medical or surgical therapy (Karimi-Abdolrezaee and Eftekharpour, 2012). Given the serious social and health implications of SCI, it is vital to study the pathophysiological mechanisms of nerve cell injury after SCI and find possible therapeutic targets and explore effective treatment options.

SCI includes primary and secondary damage (Sawada et al., 2015). The primary damage caused by direct tissue damage serves as the nidus from which the secondary mechanisms of the injury, which involve a cascade of vascular, cellular, and biochemical events, develop (Zhang et al., 2013). Secondary injury is considered to be the most destructive, as neuronal death, an important factor affecting the recovery of nerve function occurs at this stage (Tohda and Kuboyama, 2011; Borgens and Liu-Snyder, 2012; Zhang et al., 2015). Factors such as free radicals, glutamate, tumour necrosis factor (TNF), ischemia, and hypoxia cause delayed neuronal death (Oyinbo, 2011; Zhang et al., 2012). Studies have reported that anoxemia after SCI leads to tissue destruction, while hypoxia gradually induces cell death alongside inflammation (Wang et al., 2017). Other data indicate that anoxemia also can lead to a variety of vascular pathologies that lead to acute arterial occlusion, ischemia, and shock (Fleming et al., 2006; Webb et al., 2010).

Current research suggests that neuron death after SCI mainly involves necrosis and apoptosis (Tohda and Kuboyama, 2011; Zhang et al., 2012). Necroptosis is activated by TNF, which relies on the activity of the receptor-interacting protein kinase 1 (RIP1) and 3 (RIP3), and is mediated by the mixed-lineage kinase domain-like (MLKL) (Vandenabeele et al., 2010; Petrie et al., 2019). It has been noted that necroptosis activation contributes to neuronal and glial death post-SCI (Fan et al., 2015; Liu et al., 2015; Fan et al., 2016). Treatment with necrostatin-1 (Nec-1), a RIP1 kinase inhibitor, prevents cell death and improves functional outcomes after injury (Wang et al., 2014b; Liu et al., 2015). Researchers found that both RIP3 and MLKL accumulated in neurons immediately after SCI and peaked at day 1, which is consistent with neuronal necroptosis observed post-SCI (Liu et al., 2018). MLKL, a key signaling molecule in necroptosis, is a critical substrate for RIP3 (Mulay et al., 2016). The RIP1/RIP3 complex initiates programmed necrosis, followed by activation *via* phosphorylation of the mitochondrial protein MLKL, thereby causing mitochondrial dysfunction. As a new mechanism for necrosis, necroptosis and mitochondrial structural and functional damage have gained considerable attention (Rui et al., 2013).

Mitochondria are organelles that produce adenosine triphosphate (ATP) in mammalian cells. In addition to energizing cells, mitochondria also regulate the cell cycle, growth, differentiation, and apoptosis. There is cumulating evidence that mitochondrial dysfunction plays an important role in the progression of CNS diseases such as Parkinson’s disease, Alzheimer’s disease, cerebral ischemic stroke, Huntington disease, multiple sclerosis, and amyotrophic lateral sclerosis (Liao et al., 2017; Rajda et al., 2017). Furthermore, mitochondrial dysfunction also induces secondary injury and neuronal death after SCI (Beattie et al., 2002; Osellame et al., 2012).

Based on the important role of MLKL in cell damage and the potential role of mitochondrial dysfunction in SCI, our study focused on the regulation of MLKL by necrosulfonamide (NSA), which specifically blocks the MLKL, for preventing mitochondrial dysfunction after SCI. It has been shown that NSA impedes SCI by inhibiting necroptosis (Wang et al., 2018a). Zhou et al. demonstrated that NSA facilitated neuroprotection after ischemic brain injury, through the degradation of MLKL expression (Zhou et al., 2017). In the study of Wang et al., the activation of RIP3 presents as phosphorylation. The phosphorylation of RIP3 then leads to activation of its substrate MLKL, and the phosphorylated MLKL regards as the activation of MLKL (Wang et al., 2018b). We examined the protective effects of NSA in oxygen-glucose deprivation (OGD)-induced cell damage assay that replicates the pathological condition of SCI *in vitro* through RIP3 and MLKL activation (Wang et al., 2018b; Li et al., 2019; Zhang et al., 2019). We also examined the protective effects and the therapeutic window of NSA in SCI-mice. The results showed that NSA protected against a decrease in mitochondrial membrane potential (MMP), ATP, glutathione (GSH), and superoxide dismutase (SOD), and an increase in reactive oxygen species (ROS) and malonyldialdehyde (MDA). It also improved the locomotor function in SCI-mice and OGD-induced spinal neuron injury through inhibition of MLKL activation. Besides, we identified the optimal therapeutic window of the protective effects of NSA, which was within 4 h in the OGD-induced model and within 12 h in the SCI-mice model. The data showed a strong association between the suppression of MLKL and reduction in spinal cord neuronal death by improving antioxidative capacity after SCI. These findings also provide a theoretical basis for research and application of NSA in SCI therapy.

## Materials and Methods

### SCI Model and Treatment With NSA *In Vivo*

C57BL/6 mice (female, 25 ± 2 g, 8–10 weeks) were anesthetized through inhalation of 3% isoflurane with a flow rate of 1 L/min. The SCI model is described previously (Wang et al., 2019). Briefly, after making the midline skin incision and exposing the T6-T7 spinous processes, a laminectomy was performed. The laminectomy was conducted at the T6-T7 vertebral level by placing an aneurysm clip with a closing force for 1 min. The sham group underwent laminectomy. SCI was evaluated before and at 1, 2, 3, 7, 14, 21, and 28 days after injury. Following the previous reports (Liu et al., 2016; Rathkey et al., 2018), NSA (Merck, Darmstadt, Germany) was dissolved in DMSO (DMSO as the vehicle) (<0.1%) and diluted in saline. In numerous studies, intraperitoneal (i.p.) injection is often used for treatment in SCI model (Scholpa et al., 2019; Yoshizaki et al., 2019). Zhou et al. demonstrate that i.p. injection of NSA reduces necrotic cell death after ischemic brain injury (Zhou et al., 2017). NSA is injected into abdominal cavity and enters the spinal cord through the blood circulation, and we detected the inhibition of MLKL activity in the spinal cord tissues. In the dose-dependence assay, mice were immediately injected i.p. with the vehicle or NSA (1, 5, or 10 mg/kg) after surgery. In the time-dependence assay, mice were divided into five groups according to the time of i.p. injection with 5 mg/kg of NSA, and the time of administration was 15 min, 1 h, 6 h, 12 h, and 24 h after surgery, respectively.

### Measurement of Forelimb Grip Strength

Chatillon (LTCM-100, Wintop Co., Shanghai, China) was used for measuring forelimb grip strength in order to evaluate the SCI. As described in our previous report (Wang et al., 2019), mice were lifted by the tail and brought close to grip gauge crossbar in order to induce their ability to grab the bar. They were then gently pulled backward in a horizontal direction in order to pull them off the crossbar. An instrument displayed the grip forces of the two forepaws of the mice. This was repeated four times, and the average grip strength was calculated.

### The Basso Mouse Scale Assessment

The Basso Mouse Scale (BMS) measured the hind limb movements during locomotion in an open field through a 0- to 9-point scale (a high score means the better hind limb function). This assessment was performed independently by two blinded researchers over 4 min at pre-injury and 1, 2, 3, 7, 14, 21, and 28 days after SCI (n  =  9 mice/group).

### Water Content of the Spinal Cord

Spinal cord contusion was evaluated by calculating the water content of the spinal cord. The damaged spinal cord was dried at 100°C for 24 h, and the spinal cord water content was calculated using the following formula: [(wet weight—dry weight)/wet weight] × 100.

### Primary Neuron Culture and Injury Model *In Vitro*

Three-day-old C57BL/6 neonatal pups were anesthetized by isoflurane in the airtight boxes and decapitated and had their vertebral columns removed. The spinal cord was isolated and cut into 1 mm^3^-pieces, after gently peeling off the meninges. The tissue pieces were digested by 0.25% trypsin for 10 min at 37°C. The cells were re-suspended in a serum-free medium, including Neurobasal, B27, and penicillin/streptomycin (50 U/ml), adjusted at a density of 1–5×10^5^ cell/ml and seeded in 24-well plate at 37°C with 5% CO_2_. The spinal cord neurons were cultured for 10 days and then subjected to OGD stimulation. Briefly, the cells were incubated in Earle’s balanced salt solution (EBSS; Hyclone, USA) and cultured in an incubator containing 95% N_2_/5% CO_2_ for 4 h. Then, the EBSS was replaced with standard neuronal culture medium for 24 h. Cells cultured in standard neuronal culture medium in the presence of ambient 5% CO_2_ served as controls. After OGD treatment, neurons were immediately treated with either NSA (Merck, Darmstadt, Germany) or Nec-1 (Sigma, St. Louis, MO, USA). The vehicle group received an equal volume of DMSO.

### Cell Viability Detection

Cell damage was measured using the CellTiter 96^®^ AQueous One Solution Cell Proliferation Assay (Promega, Madison, WI, USA). Cells were plated in 96-well plates in triplicate at approximately 3×10^4^ cells per well and cultured in the growth medium. After treatment, MTS was added to the culture medium and incubated for 2 h at 37°C in humidified 95% air and 5% CO_2_. The absorbance was measured at 450 nm using a microplate reader (Thermo Scientific, Waltham, MA, USA).

### ATP Measurement

ATP was detected through an ATP Content Assay Kit (Solarbio, Beijing, China) following the manufacturer’s instructions. Cells were ultrasonically disrupted and centrifuged at 800 × g for 10 min at 4°C. Creatine kinase catalyses the reaction between creatine and ATP to produce phosphocreatine. To detect the ATP level, the content of phosphocreatine was determined by the colorimetric method of phosphomolybdic acid. The absorbance at 700 nm was measured by a microplate reader (Thermo Scientific).

### MMP Measurement

MMP was detected with JC-1 (Solarbio); 20 µl of JC-1 solution (0.15 mM in DMSO) was added to 100 μl of each suspension and was incubated at 37°C for 20 min; then, JC-1 monomer was detected at 490 nm excitation and 530 nm emission wavelengths, and JC-1 polymer was detected at 5,250 nm excitation and 590 nm emission wavelengths using a fluorescence spectrophotometer (Thermo Scientific).

### Measurement of the antioxidative Capacity

The activities of SOD, MDA, and GSH were detected according to the manufacturer’s protocol (Nanjing Jiancheng Bioengineering Institute, Nanjing, China). Homogenate was obtained from tissue or cell, centrifuged for 10 min at 10,000 × g at 4°C, and the supernatant was stored at −80°C. For SOD detection, the reaction system contained 20 µl WST-1 enzyme reaction solution, 20 µl sample, and 200 µl enzyme substrate. The reaction mixtures were incubated at 37°C for 20 min. One unit of SOD activity was defined as the amount of enzyme required to cause 50% inhibition of the reduction of WST-1 formazan detected at 450 nm. For MDA detection, 20 µl of the sample and 200 µl of thiobarbituric acid reaction solution were mixed and heated at 95°C for 40 min. After cooling, the supernatant was obtained by centrifugation at 4,000 rpm for 10 min, and MDA concentration was detected in the supernatant at 532 nm. For GSH detection, erythrocytes from mouse whole blood or cells treated *in vitro* were collected and lysed; then, 100 µl of the supernatant, 100 µl of oxidized glutathione solution, and 20 µl of NADPH solution (6 mM) were mixed, and GSH was detected in the supernatant at 405 nm.

The ROS detection was performed according to the manufacturer’s instructions (Nanjing Jiancheng Bioengineering Institute). Following the indicated treatment, the monoplast suspensions were harvested and then, resuspended in 10 μM 2′, 7′-dichlorofluorescein diacetate (DCFH-DA) solution and incubated in a CO2 incubator at 37°C for 30 min in the dark, followed by washing with PBS for three times. Finally, the cells were resuspended in 0.5 ml of PBS, and the fluorescence intensity of the samples was analysed using a fluorescence microplate.

### Quantitative Real-Time PCR Analysis

The total RNA was extracted from samples using TRIzol reagent (Solarbio). Total RNA was reversely transcribed to cDNA using a HiFi-MMLV cDNA First Strand Synthesis Kit (CW Bio, Beijing, China). Quantitative real-time PCR was performed using GoTaq qPCR Master Mix (Promega) on the CFX96TM Real-Time System (Bio-Rad, Hercules, CA). Primer sequences were as follows: MLKL: F: 5´ -ACCCTTCAGAGGCACAACAC- 3´, R: 5´ -TGTCATTGGATTCGGTGGGG-3´; GAPDH: F: 5´ -AACTTTGGCATTGTGGAAGG- 3´, R: 5´ -GGATGCAGGGATGATGTTCT- 3´.

### Western Blot Analysis

Protein was harvested in 1×lysis buffer including a protease inhibitor. 30 µg total protein was separated by 10% SDS-PAGE, transferred to PVDF membrane, blocked for 1 h with TBST containing 5% BSA, and incubated at 4 °C overnight with the appropriate primary antibodies: RIP3 (Abcam, ab56164, 1:1,000), pRIP3 (Abcam, ab222320, 1:1,000), MLKL (CST, #37705, 1:1,00), p-MLKL (CST, #37333, 1:10,00), Bax (Abcam, ab32503, 1:1,000), Bcl-2 (Abcam, ab32124, 1:1,000), and GAPDH (Abcam, ab181602, 1:20,000). They were then incubated with HRP-conjugated secondary antibodies (Abgent, 1:30,000), which was followed by chemiluminescent substrate development (Bio-Rad).

### Neun and MLKL Double Immunofluorescence Staining

Cells were fixed with 4% paraformaldehyde for 15 min at 37°C, washed three times with PBS, and then, incubated overnight at 4°C in PBS containing 2% Goat serum, 0.03% Triton X-100 in, p-MLKL antibody (CST, #37333, 1:1,200), and NeuN antibody (Abcam, ab177487, 1:500). They were then incubated for 2 h at 25°C with the following fluorescent secondary antibodies. Cell nuclei were stained with DAPI, and images were obtained using a fluorescence microscope (Olympus, Osaka, Japan).

### TUNEL Staining

Spinal cord neurons were harvested, and the damage was determined by DeadEnd™ Fluorometric Terminal deoxynucleotidyl transferase dUTP nick end labeling (TUNEL) System (Promega). TUNEL staining was conducted with fluorescein-dUTP for apoptotic cell nuclei and DAPI stain for all cell nuclei. Images were obtained using a fluorescence microscope (Olympus).

### Statistical Analysis

All statistical analyses were performed with SPSS 22.0 and analysed using the Student’s *t*-test or a one-way ANOVA, followed by Tukey’s multiple comparison test, with *p* < 0.05 considered statistically significant. Each experiment consisted of at least four replicates per condition. All data are described as the mean ± SEM.

## Results

### Cytoprotective Effect of NSA After OGD

**Figure 1:**
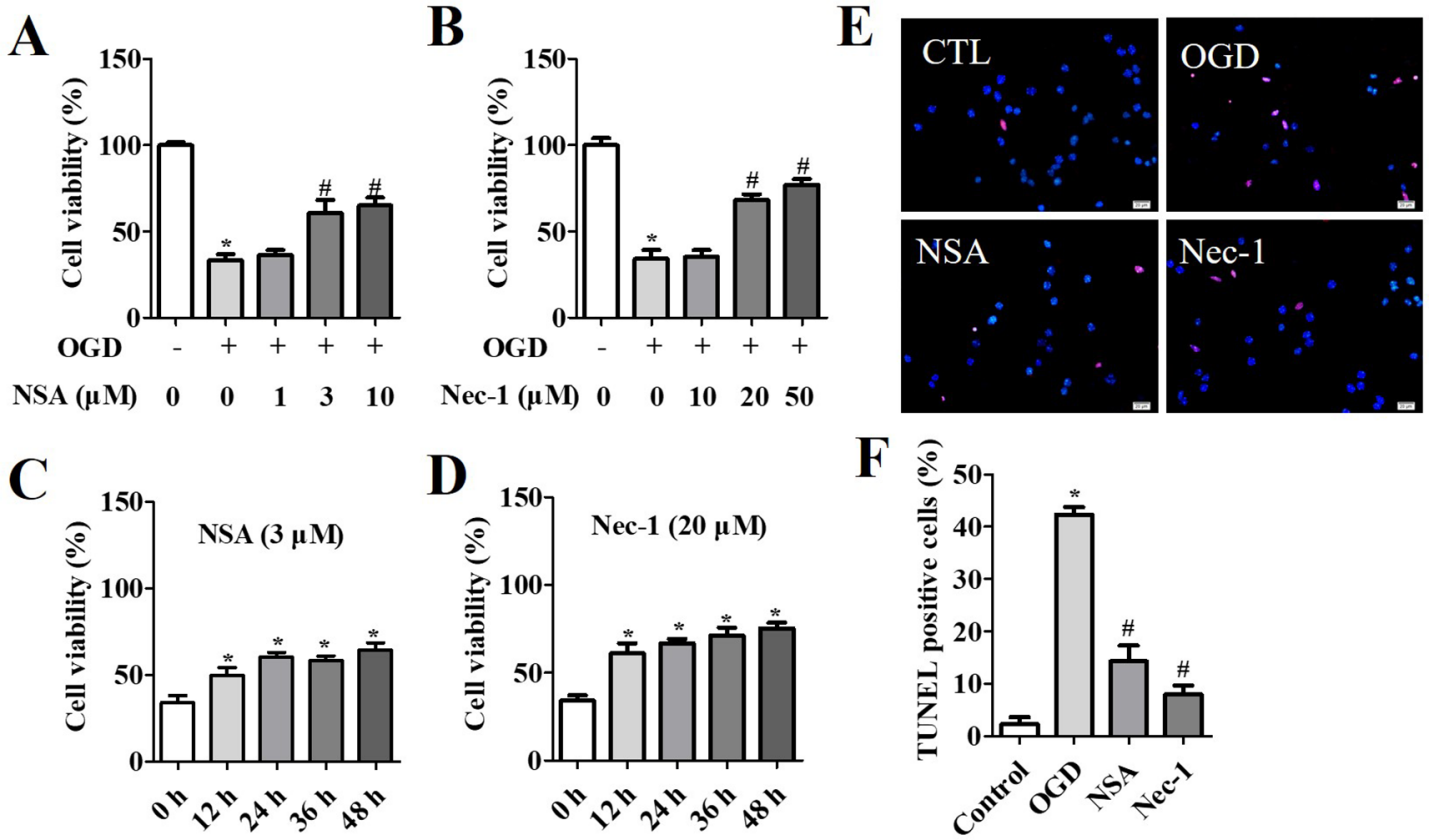
*Cytoprotection of NSA in OGD-induced spinal cord neurons.* Cell viability after NSA treatment (1, 3, and 10 μM) **(A)** and Nec-1 treatment (10, 20, and 50 μM) **(B)** for 24 h after OGD treatment. Cell viability after 3 μM Nec-1 treatment **(C)** and 20 μM Nec-1 treatment **(D)** for 12, 24, 36, and 48 h before OGD treatment, respectively. **(E)** TUNEL staining was performed for cell damage and DAPI stained all cell nuclei. **(F)** Histogram analysis of TUNEL positive cell rata. Scale bars: 50 μm. Data are presented as the mean ± SEM, n = 4. *p < 0.05, *vs.* the control group (CTL). ^#^ *p* < 0.05, *vs.* the OGD group. **(A, B** and **F)**: Data were analyzed by one-way ANOVA followed by Tukey’s multiple comparisons tests. **(C** and **D)**: Data were analyzed using Student’s t-test. OGD, oxygen-glucose deprivation; NSA, necrosulfonamide; Nec-1, necrostatin-1; ATP, adenosine triphosphate;.

NSA specifically blocks MLKL, while Nec-1 is an inhibitor of RIP1. Therefore, they act as inhibitors of necroptosis. In the OGD-induced assay, the viability of NSA-treated cells at 3 μM and 10 μM (**Figure 1A**) and Nec-1-treated cells at 20 μM and 50 μM (**Figure 1B**) was significantly increased compared to that of cells in vehicle groups. The cell survival rate was measured after NSA (3 μM) or Nec-1 (20 μM) treatment at 12 h, 24 h, 36 h, and 48 h. We observed a significant time-dependent reversion of cell viability with NSA (**Figure 1C**) and Nec-1 (**Figure 1D**) treatment. TUNEL staining was conducted in order to investigate the role of NSA treatment in cell death (**Figures 1E, F**). OGD induced the death of most cells compared to controls, and NSA treatment reduced the TUNEL positive cell rate. The results indicate that NSA treatment can ameliorate spinal cord neuronal death.

### The Effect of NSA on Mitochondrial Dysfunction Through Inhibiting p-MLKL

**Figure 2:**
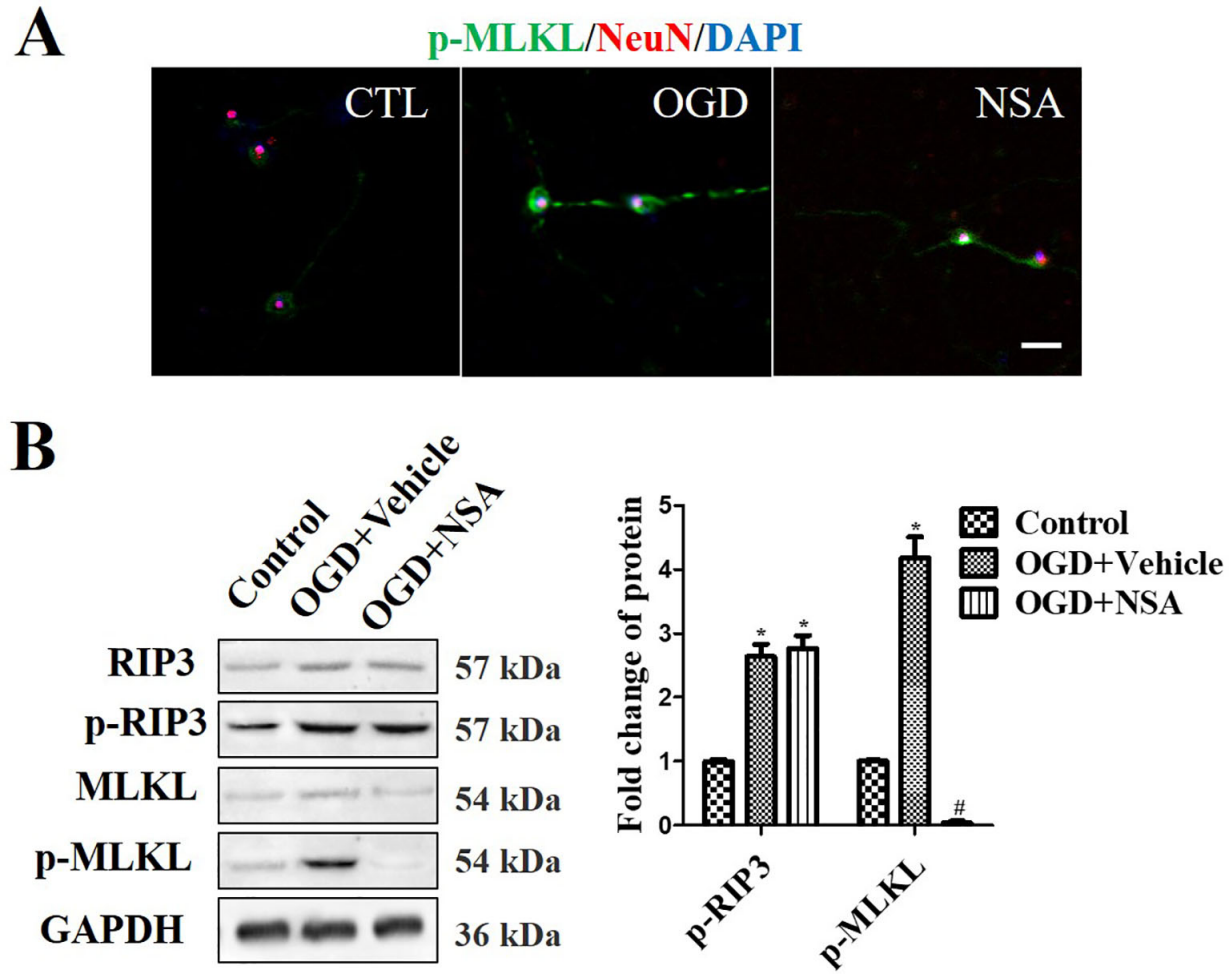
*NSA suppresses MLKL activation but not p-RIP3.* Prevention of MLKL activation by treatment with 3 μM NSA for 24 h after OGD treatment. **(A)** NeuN and p-MLKL double staining. Cell nuclei were stained with DAPI (blue fluorescence) and neurons were stained with NeuN (red fluorescence). Pictures were taken using a fluorescence microscope (scale bar = 20 μm). **(B)** WB analysis of the level of p-RIP3 and p-MLKL induced by OGD, p-MLKL level was significantly reversed after NSA treatment. Data are presented as the mean ± SEM, n = 4. *p < 0.05, *vs.* the control group (CTL). ^#^ *p* < 0.05, *vs.* the OGD group. Data were analyzed using Student’s t-test. RIP3, receptor interacting protein kinase-3; OGD, oxygen-glucose deprivation; NSA, necrosulfonamide; MLKL, mixed-lineage kinase domain-like protein; Nec-1, necrostatin-1; WB, Western blot.

Western Blot (WB) analysis and immunoﬂuorescence analyses were performed to assess the role of MLKL activity (p-MLKL level) in OGD-induced damage. NeuN and p-MLKL double staining was conducted in order to observe the effect of NSA on MLKL activation in spinal cord neurons. The results revealed that OGD at 4 h induced p-MLKL, while NSA relieved MLKL activation but not RIP3 activation (**Figure 2A**). Meanwhile, in WB analysis, the enhanced protein level of p-MLKL induced by OGD was significantly reduced due to NSA treatment (*p* < 0.05); however, NSA did not reduce OGD-induced high levels of p-RIP3 (**Figure 2B**).

**Figure 3:**
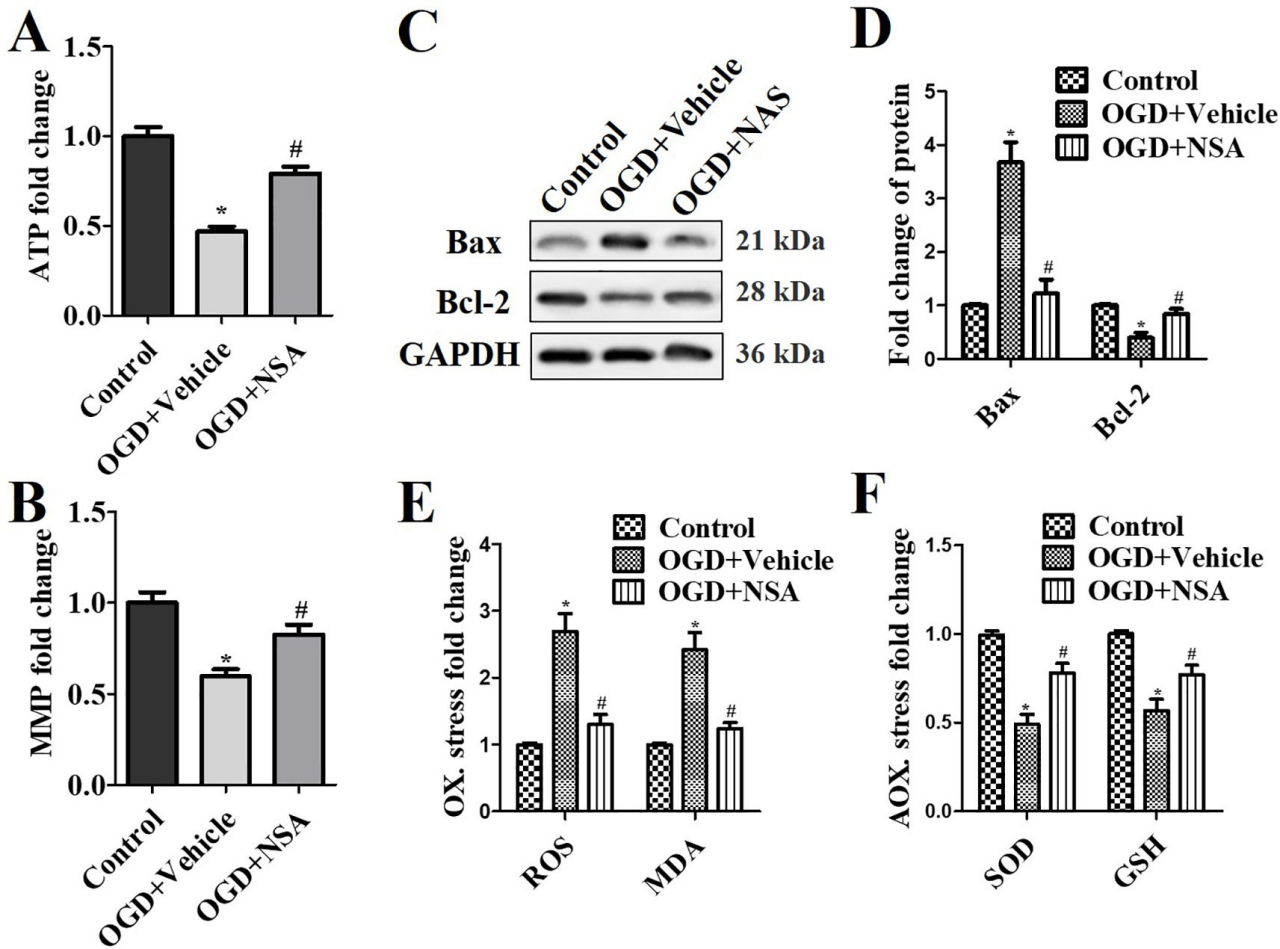
*NSA treatment ameliorates mitochondrial dysfunction and neuronal death.* After 3 μM NSA treatment for 24 h after OGD treatment, the effect of NSA treatment on ATP level **(A)**, MMP **(B)**, Bax and Bcl-2 expression **(C** and **D)**, the oxidative stress ROS and MDA levels **(E)**, and antioxidative capacity SOD and GSH levels **(F)** were detected in OGD-induced spinal cord neuron. Data are presented as the mean ± SEM, n = 4. *p < 0.05, *vs.* the control group (CTL). ^#^*p* < 0.05, *vs.* the OGD group. A and B: Data were analyzed by two-way ANOVA followed by Tukey’s multiple comparison test. C: Data were analyzed by one-way ANOVA followed by Tukey’s multiple comparison test. OGD, oxygen-glucose deprivation; NSA, necrosulfonamide; ATP, adenosine triphosphate; MMP, mitochondrial membrane potential; GSH, glutathione; SOD, superoxide dismutase; ROS, reactive oxygen species; MDA, malonyldialdehyde.

As shown in **Figure 3**, the role of NSA in mitochondrial capacity and antioxidative capacity were also assessed *in vitro*. After OGD treatment, the ATP and MMP levels were reduced by 50% and 40%, respectively; however, NSA significantly protected the mitochondrial integrity and recovered the levels of ATP and MMP (*p* < 0.05, **Figures 3A, B**). Bax is a pro-apoptotic member of the Bcl-2 protein family and exists in the outer mitochondrial membrane (Jurgensmeier et al., 1998). As an inner mitochondrial membrane protein, Bcl-2 inhibits programmed cell death (Hockenbery et al., 1990). Bax and Bcl-2 are regarded as possible indicators of mitochondrial dysfunction. OGD induced Bax expression and suppressed Bcl-2 levels, and NSA improved the expression of Bax and Bcl-2 (*p* < 0.05, **Figures 3C, D**). The suppressed Bax and up-regulated Bcl-2 demonstrated the protective role in mitochondrial function by NSA in OGD-stimulated spinal cord neurons. The antioxidative capacity was assessed after NSA incubation to analyse the effect of NSA on the extent of intracellular oxidative stress. OGD increased the levels of the ROS and MDA (*p* < 0.05, **Figure 3E**) and reduced the levels of the SOD and GSH (*p* < 0.05, **Figure 3F**) in comparison with controls. NSA also increased mitochondrial capacity and antioxidative capacity compared with the OGD group (*p* < 0.05, **Figures 3E, F**).

### NSA Improves the Motor Function and Spinal Edema of SCI-Mice With a Therapeutic Window

**Figure 4:**
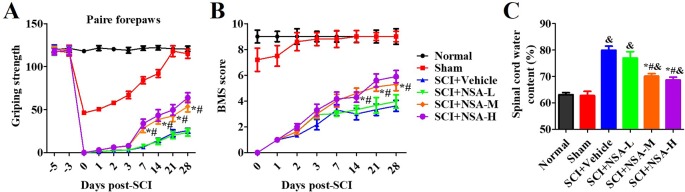
*NSA improves the motor function and spinal edema of SCI-mice.* The behavioral performances in mice with SCI or treatment with NSA (L: 1 mg/kg, M: 5 mg/kg, H: 10 mg/kg) were shown at 1, 2, 3, 7, 14, 21, and 28 days following surgery. **(A)** pair forepaws were measured. **(B)** Daily measurement of the BMS scores. **(C)** Dry and wet specific gravity method was used to measure spinal edema at 3 days post-SCI. Data are reported as the mean ± SEM, n = 9. ^&^ *p <* 0.05, *vs.* the sham group. *p < 0.05, *vs.* the SCI+Vehicle group. ^#^ *p <* 0.05, *vs.* the NSA-L group. A and B: Data were analyzed by two-way ANOVA followed by Tukey’s multiple comparison test. C: Data were analyzed by one-way ANOVA followed by Tukey’s multiple comparison test. SCI, spinal cord injury; NSA, necrosulfonamide; BMS, basso mouse scale.

As shown in **Figure 4**, one section of mice was injected with a vehicle or NSA (1, 5, or 10 mg/kg i.p.), immediately after surgery. The behavioural performances assessed by the forelimb grip strength test and BMS score system were measured at 1, 3, 7, 14, 21, and 28 days post-SCI. The grip strength provisionally decreased in the sham group after surgery but returned to its preoperative levels within 7 days. SCI induced serious motor disfunction, such as the decreased grip strength of forelimbs and hind limbs. Although this phenomenon was resolved by 7 to 28 days, it still appeared as a significant dysfunction (**Figure 4A**). In the previous week, treatment with NSA did not ameliorate the power of grip force and dyskinesia. However, the forepaw function of NSA-treated mice began to significantly improve from the 7^th^ day onwards in the 5 mg/kg- and 10 mg/kg-treated groups compared to the SCI group (*p* < 0.05, **Figure 4A**). As shown in **Figure 4B**, a similar effect of NSA was observed in the forelimb grip strength test and BMS score, and 5 mg/kg and 10 mg/kg NSA groups showed the same protective function after SCI (**Figure 4B**).

**Figure 5:**
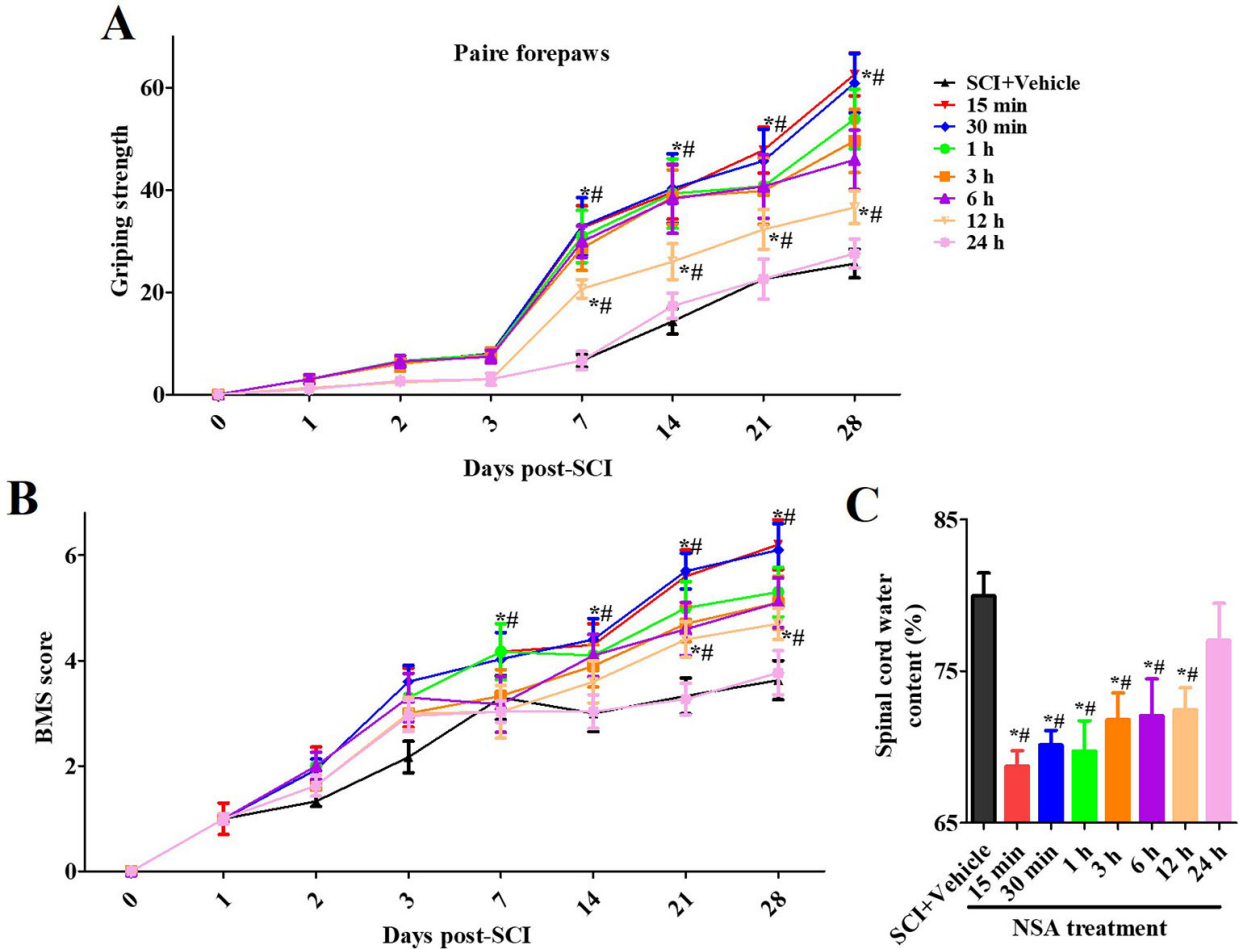
*The therapeutic window of NSA after SCI.* The time of administration of NSA was 15 min, 30 min, 1 h, 3 h, 6 h, 12 h, and 24 h after surgery in groups, respectively. The behavioral performances conducted by forelimb grip strength test **(A)** and BMS score system **(B)** were observed at 1, 3, 7, 14, 21, and 28 days following SCI surgery. **(C)** Measurement of spinal edema at 3 days post-SCI. Data are reported as the mean ± SEM, n = 9. *p <0.05, *vs.* the SCI+Vehicle group. ^#^ *p <* 0.05, *vs.* the 24 h group. **(A** and **B)**: Data were analyzed by two-way ANOVA followed by Tukey’s multiple comparison test. C: Data were analyzed by one-way ANOVA followed by Tukey’s multiple comparison test. SCI, spinal cord injury; NSA, necrosulfonamide; BMS, basso mouse scale.

We also confirmed the effects of NSA treatment on spinal cord oedema of SCI-mice. The water content of the spinal cord was significantly enhanced post-SCI; however, it was found reduced in 5 mg/kg and 10 mg/kg NSA groups, but not in the 1 mg/kg NSA group (*p* < 0.05, **Figure 4C**). Another section of SCI-mice was divided into groups according to the time of administration of NSA (5 mg/kg i.p.), and the times of administration of NSA were 15 min, 30 min, 1 h, 3 h, 6 h, 12 h, and 24 h after surgery. Forepaw function of NSA-treated mice began to significantly improve from the 7^th^ day onwards post-SCI in 15 min, 30 min, 1 h, 3 h, 6 h, and 12 h groups, and the grip strength ameliorated with time compared to the SCI group (*p* < 0.05, **Figure 5A**). There were no significant improvements in the 24 h group. The 15 min and 30 min groups demonstrated the best improvement effect, and the later the NSA administered, the slower the grip strength recovered (*p* < 0.05, **Figure 5A**). As shown in **Figure 5B**, the same time trend of NSA was observed in BMS score and oedema. 15 min and 30 min groups displayed a better protective effect during 7 to 28 days (*p* < 0.05, **Figure 5B**). The BMS scores in 1 h, 3 h, 6 h, and 12 h groups still improved significantly from 21 to 28 days (*p* < 0.05, **Figure 5B**). As shown in **Figure 5C**, the 24 h group revealed a weak effect on spinal oedema, while oedema was significantly decreased in 15 min, 30 min, 1 h, 3 h, 6 h, and 12 h groups (*p* < 0.05, **Figure 5C**). The data suggest that NSA relieves the spinal cord damage and improves the mobility of SCI-mice. This effect was closely related to the time of administration of NSA, and the administration within 12 h of SCI had a better improvement rate. In conclusion, NSA displayed a protective effect in an optimal therapeutic window, which is within 12 h, in the SCI-mice model.

### NSA Reverses Mitochondrial Capacity and Antioxidative Capacity *via* Inhibiting MLKL Activation *In Vivo*

**Figure 6:**
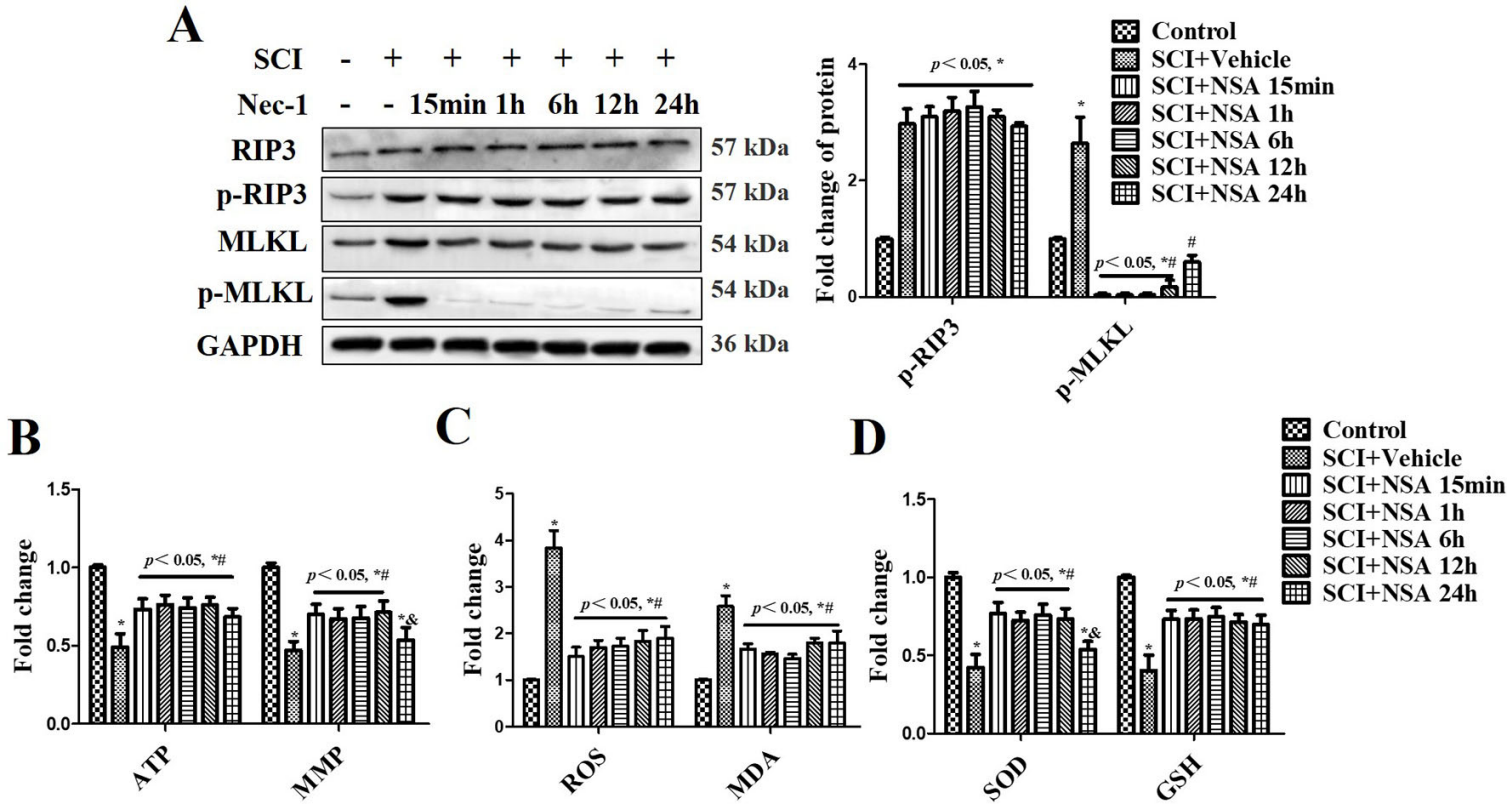
*NSA reverses mitochondrial capacity and antioxidative capacity via inhibiting MLKL activation in spinal cord tissues.* After SCI, NSA was treated at different times, such as 15 min, 1 h, 6 h, 12 h, and 24 h. **(A)** The protein expression of p-MLKL was detected at 3 days post-SCI using WB analysis, histogram analysis of change of p-MLKL. ELISA analysis of mitochondrial dysfunction at 3 days post-SCI, including ATP and MMP levels **(B)**, and the unbalanced antioxidant capacity [the levels of ROS and MDA **(C)** and the levels of SOD and GSH **(D)**]. Data are reported as the mean ± SEM, n = 8. *p < 0.05, *vs.* the sham group. ^#^ *p <* 0.05, *vs.* the SCI+Vehicle group. ^&^ *p <* 0.05, *vs.* the SCI+NSA 15-min group. Data were analyzed by one-way ANOVA followed by Tukey’s multiple comparison test. SCI, spinal cord injury; NSA, necrosulfonamide; MLKL, mixed-lineage kinase domain-like protein; ATP, adenosine triphosphate; MMP, mitochondrial membrane potential; GSH, glutathione; SOD, superoxide dismutase; ROS, reactive oxygen species; MDA, malonyldialdehyde; WB, Western blot.

The levels of p-RIP3 and p-MLKL were measured at 3 days after SCI (**Figure 6A**). p-RIP3 and p-MLKL levels significantly increased at 3 days after SCI compared to the sham group (*p* < 0.05). In NSA-treated groups (at 15 min, 1 h, 6 h, and 12 h after SCI), p-MLKL expression was reduced after SCI; however, it was still high in the NSA-treated group at 24 h (*p* < 0.05, **Figure 6A**). p-RIP3 levels remained unchanged after NSA treatment in SCI-mice (**Figure 6A**). SCI induced mitochondrial dysfunction at 3 days post-SCI, including the reduction of ATP, MMP, and the abnormal antioxidant capacity (**Figures 6B–D**). Treatment with NSA at 15 min, 1 h, 6 h, and 12 h after SCI significantly ameliorated the ATP and MMP levels (**Figure 6B**, *p* < 0.05, respectively). Redox status measurements were conducted in each group. SCI led to an increase in ROS and MDA levels and a decrease in SOD and GSH levels compared to the sham group (**Figures 6C, D**, *p* < 0.05, respectively). NSA treatment (in 15 min, 1 h, 6 h, and 12 h groups) resulted in a significant reduction in ROS and MDA levels and an increase in SOD and GSH levels compared to the SCI group (**Figures 6C, D**, *p* < 0.05, respectively). The effect on MMP and SOD was weaker in the 24 h group than that in the other NSA groups, but the 24 h group still revealed significant improvement in the ATP, ROS, MDA, and GSH levels compared to the SCI + vehicle group (**Figures 6B–D**, *p* < 0.05, respectively). Therefore, NSA demonstrated a more superior effect on mitochondrial dysfunction, including improvements in mitochondrial integrity and antioxidant capacity, within 12 h after SCI compared to the treatment at 24 h.

**Figure 7:**
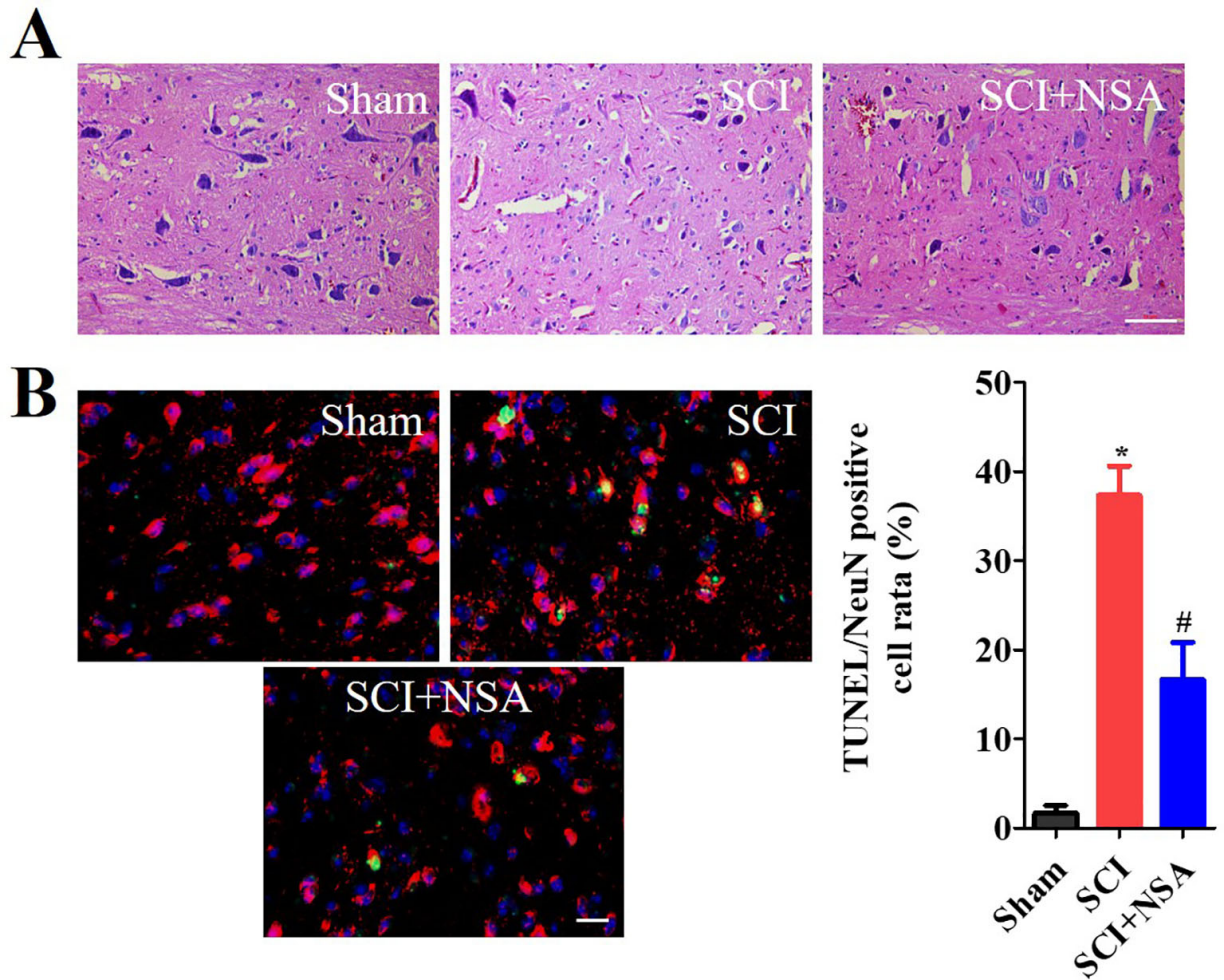
*NSA improves the recovery of SCI and the survival of neurons.* **(A)** HE staining results of the sham, SCI and SCI+NSA group. Treated with 5 mg/kg NSA 15 min after SCI, NSA significant protective effect with less necrosis, karyopyknosis, infiltrated macrophages compared with the SCI group, scale bar = 50 μm. **(B)** NeuN/TUNEL staining results of the sham, SCI and SCI+NSA group, scale bar = 20 μm. Analysis of the positive neurons of the TUNEL staining results. *p < 0.05 *vs.* the sham group, ^#^ *p*  < 0.01 *vs.* the SCI group. n  =  5. SCI, spinal cord injury; NSA, necrosulfonamide; TUNEL, terminal deoxynucleotidyl transferase-dUTP nick end labeling.

As shown in **Figure 7A**, HE staining results of the spinal cord showed that the structure of the spinal cord was clear and the nucleus was complete and inerratic, and SCI induced progressive destruction of the vacuoles and sparse reticular changes of the white matter, extensive denatured neurons, and nuclear condensation, fragmentation and dissolution. NSA treatment resulted in a significant protective effect with less necrosis, karyopyknosis, and infiltration by macrophages compared with the SCI group. To further confirm the protective effect of the NSA, we investigated the survival of neurons directly by NeuN/TUNEL staining. As shown in **Figure 7B**, TUNEL positive cells were significantly increased after SCI, while NSA protected against the increase in NeuN/TUNEL positive cells (*p* < 0.0001). The data indicated that NSA protected against neuronal damage and promoted recovery post-SCI.

## Discussion

Due to the limited ability of the CNS to repair itself following injury, SCI is considered an irreversible disease that causes plegia, with functional deficits (Courtine and Sofroniew, 2019). Surgical procedures, supportive measures, and rehabilitation protocols have improved functional outcomes and decreased morbidity in patients with SCI (Fehlings et al., 2017). However, to date, no randomized clinical trial has demonstrated the efficacy of a repair strategy for improving functional recovery from SCI (Courtine and Sofroniew, 2019). Secondary injury after primary trauma may explain the demyelination and loss of neural circuits and the progress of many neurologic deficits post-SCI (Ahuja et al., 2017). The delay in secondary injury provides a window for therapeutic intervention in order to improve functional recovery and prevent further tissue damage. Spinal cord compression results in ischemia and hypoxia, and the degree of ischemia and hypoxia is proportional to the degree of neurological impairment (Ellingson, 2019). OGD stimulation is a well-established model of ischemia *in vitro* (Wang et al., 2011; Wang et al., 2012a). In our research, we used the OGD-induced injury model *in vitro* to simulate the state of hypoxia after SCI. As mentioned previously, mitochondrial health and function are vital for sustaining cellular biological energy requirements, and mitochondrial dysfunction has been considered as a crucial contributor to the progress of human diseases, including CNS diseases. Mitochondrial dysfunction is often associated with increased oxidative stress, which leads to cell damage and death (Chen et al., 2016). We examined the protective effects of NSA in OGD-induced cell damage. We also investigated the protective effects and the therapeutic window of NSA in SCI-mice. The results showed that NSA protected against a decrease in MMP, ATP, GSH, and SOD levels and an increase in ROS and MDA levels. NSA also improved the locomotor function in SCI-mice and OGD-induced spinal neuron injury through the inhibition of MLKL activation. In addition, the protective effects of NSA demonstrated a therapeutic window. The optimal treatment time was within 12 h in the SCI-mice model.

Some studies suggest that suppressing necroptosis may be a valid therapeutic strategy for protecting cell viability and neurologic functions after injury (Wang et al., 2014b; Zhou et al., 2017; Wang et al., 2018a). Activities of RIP1 and RIP3 and their interaction with MLKL are necessary for TNF-α-induced necroptosis (Deng et al., 2019; Lin et al., 2019; Yuan et al., 2019). It has been demonstrated that RIP1- and RIP3-containing protein complexes are formed specifically in response to necrosis induction (Wang et al., 2012b). MLKL inhibits necroptosis after being recruited and phosphorylated by RIP3 (Wang et al., 2014a), which activates PGAM5/Drp1 leading to mitochondrial fragmentation, an early and mandatory step of necrosis (Lee et al., 2004; Jagasia et al., 2005; Breckenridge et al., 2008). MLKL has been shown to translocate to the mitochondrial fraction upon stimulation in multiple cell types, indicating that ROS production can be a vital step in the inhibition of the necroptotic progress (Wang et al., 2012b; Marshall and Baines, 2014), and impaired regulation of mitochondrial dynamic proteins contributes to ischemic injury *via* reducing energy production and promoting ROS generation (Calo et al., 2013). Zhu et al. verified that NS309 significantly improved hind-limb motor function scores at 72 h of SCI/R challenged rabbits through anti-oxidative activity and inhibition of mitochondrial dysfunction. In traumatic SCI, Natrium Benzoate alleviates neuronal apoptosis *via* the DJ-1-related anti-oxidative stress pathway involving Akt phosphorylation (Gao et al., 2019). The calcium-sensing receptor (CaSR) has been found after spinal cord ischemia-reperfusion injury. The CaSR can mediate the overload of intracellular calcium ions, leading to mitochondrial damage, which is characterized by the opening of mitochondrial permeability transition pores, reduced ATP production, and ultimately, activation of downstream caspase-3 leading to apoptosis (Nakamura et al., 2000; Lu et al., 2015). It has been demonstrated that there are causal relationships between the recovery of motor function and anti-oxidative activities. This is consistent with our study findings that MLKL content increased significantly post-SCI and NSA improved recovery of neurological function *via* the MLKL-related anti-oxidative stress pathway.

OGD disrupts mitochondrial membranes, and severe energy deficiency leads to MMP consumption, resulting in necrosis (Almeida et al., 2002; Iijima, 2006). The interaction of RIP3 with MLKL has been shown to promote translocation of the RIP1/RIP3/MLKL complex to the mitochondrial related membrane fraction of cells, that is, the contact sites between the outer mitochondrial membrane and ER membrane (Chen et al., 2013). Moreover, MLKL oligomerizes and then, executes programmed necrosis (Cai and Liu, 2018). Qu et al. demonstrated that MLKL oligomerization was increased in the membrane fraction of the OPCs after an OGD/zVAD insult (Qu et al., 2017). Qu et al. demonstrated that MLKL siRNA decreased RIP1-RIP3-MLKL interaction and attenuated OGD/zVAD-induced neuron death (Qu et al., 2016). The translocation of oligomerized MLKL to the neuronal membrane resulting in the damage of the cellular membrane is a possible new mechanism of neuron necroptosis. MLKL inhibition attenuates hypoxia-ischemia-induced brain injury in neonatal rats *in vivo* (Qu et al., 2016). The specific blockade of the MLKL by NSA confirms the protective effect of the NSA (Dong et al., 2017; Wang et al., 2018a). Zhou et al. demonstrated that NSA facilitated neuroprotection after ischemic brain injury through the inhibition of MLKL expression (Zhou et al., 2017). It is reported that Nec-1 suppresses necroptosis post-SCI and protects neurons *via* reducing vacuolar degeneration (Wang et al., 2014b). However, the effect of NSA on mitochondrial dysfunction post-SCI has been rarely reported. Although Wang et al. showed that NSA alleviated SCI by suppressing necroptosis, the specific mechanism of action and the treatment window of NSA deserves further discussion (Wang et al., 2018a). In this study, we found that the NSA showed a cytoprotective effect after SCI. Meanwhile, NSA treatment could improve antioxidative capacity and motor function after SCI. It is worth mentioning that in order to be clinically applicable, NSA is to be delivered at different time points after SCI *in vivo*. The optimal treatment time of NSA is within 12 h of injury in the SCI-mice model, which is related to the improvement of antioxidative capacity. Administering this agent as soon as possible may help to alleviate neuronal damage and might be related to the delayed nature of the secondary injury.

In this study, considering that MLKL is a pivotal regulator of necrosis signalling downstream of RIP3, we measured the MLKL activation after OGD and SCI. However, there is evidence that indicates that RIP3 and MLKL do not always act synergistically. Their respective genetically deleted, organ injury models of mice demonstrate differential phenotypes (Newton et al., 2016). According to Newton et al. (2016), MLKL deficiency improves survival following kidney ischaemia-reperfusion injury, loss of MLKL provides significant protection against TNF (500 μg/kg body weight)-induced body temperature increase, and MLKL deficiency weakly regulates the increase in G-CSF, IL-6, and CXCL1 levels. RIP3 also exerts an effect on kidney function *via* mitochondrial dysfunction independently of MLKL (Sureshbabu et al., 2018). Gutierrez et al. verified that MLKL activation triggered potassium efflux and assembly of the NLRP3 inflammasome, which processes and activates IL-1β during necroptosis, and MLKL activation also caused cell membrane disruption to allow the efficient release of IL-1β (Gutierrez et al., 2017). Zhang et al. demonstrated that MLKL deficiency prevented DSS-induced inflammatory cytokines production, MAPK signalling activation, and colitis (Zhang et al., 2019). Lin et al. showed that RIP3 or MLKL deletion improved MPTP-induced neuroinflammation (including TNF-α, IL-1β, and IL-6 levels) and DA neuron necroptosis (Lin et al., 2019). According to Newton et al., RIPK1 and RIPK3 regulate more than just MLKL-dependent necroptosis (Newton et al., 2016). Moreover, the function of the p-MLKL S441 is distinct from the necroptosis-inducing phosphorylation by RIP3 kinase (Ying et al., 2018). Thus, the functions of RIP3 and MLKL may vary with varying degrees of injury or different animal models. We demonstrated NSA ameliorated neurological impairment in SCI by inhibiting MLKL-dependent necroptosis. However, the synergistic or antagonistic regulation of a series of factors, such as RIP3, MLKL, necroptosis, and mitochondrial function, should be a possible future avenue of research.

In summary, the mitochondrial hypothesis implicates an earlier mechanism of neuronal death after SCI, that may allow for a therapeutic window for neuroprotective intervention. The optimal treatment time of NSA was found to be within 12 h in the SCI-mice model, which was closely related to the improvement of antioxidative capacity independent of RIP3 phosphorylation. However, much remains to be elucidated regarding the role of NSA/MLKL in mitochondrial dysfunction involving neuronal necroptosis. Further investigations are required in order to confirm the intrinsic mechanisms and functions of NSA in mediating necroptosis after SCI.

## Data Availability Statement

All datasets generated for this study are included in the article.

## Ethics Statement

This study was carried out in accordance with the principles of the Basel Declaration and recommendations of the NIH Guide for the Care and Use of Laboratory Animals. The protocol was approved by the Animal Care and Use Committee of the Jilin University.

## Author Contributions

JJ and MW contributed to the conception and design of the research, interpreted the results of the experiments, and edited and revised the manuscript. YW and PR performed the experiments. JJ and SS prepared the figures and analyzed the data. JJ drafted the manuscript.

## Funding

The Science and Technology Development Plan Project of Jilin Province (No. 20180520128JH) and the Specific Talent Project for Medical and Health of Jilin Province (2019SCZT031) supported this work.

## Conflict of Interest

The authors declare that the research was conducted in the absence of any commercial or financial relationships that could be construed as a potential conflict of interest.

## Abbreviations

SCI, spinal cord injury; CNS, central nervous system; TNF, tumor necrosis factor; RIP3, receptor interacting protein kinase-3; OGD, oxygen-glucose deprivation; NSA, necrosulfonamide; MLKL, mixed-lineage kinase domain-like protein; Nec-1, necrostatin-1; ATP, adenosine triphosphate; MMP, mitochondrial membrane potential; GSH, glutathione; SOD, superoxide dismutase; ROS, reactive oxygen species; MDA, malonyldialdehyde; EBSS, Earle’s balanced salt solution; BMS, basso mouse scale; TUNEL, terminal deoxynucleotidyl transferase-dUTP nick end labeling; CaSR, calcium sensing receptor.
